# Natrum Sul. in Asthma

**Published:** 1887-04

**Authors:** Wm. Jefferson Guernsey

**Affiliations:** Philadelphia


### NATRUM SUL. IN ASTHMA.

WM. JEFFERSON GUERNSEY, M. D., PHILADELPHIA.

In the August (1886) number of THE HOMŒOPATHIC PHYSICIAN appeared some notes from a lecture on Nat. sul. by Professor J. T. Kent, wherein he refers (p. 278) to the value of this remedy in asthma. I have recently had two opportunities of verifying it, and take pleasure in tendering this bit of evidence as corroborative of that excellent authority.

I. E. G. S., female, married, aged thirty-six years. Attack violent; greenish, purulent expectoration ; a loose evacuation immediately after rising for last two days. ℞. Nat. sul.$^{500}$ (Tafel) in water every two hours. She was enabled to lie down that night; respiration and cough much improved and the expectoration easier. The next day she was so much better that she ceased taking the medicine before I called, less than twenty-four hours from first visit.

II. C. C., female, married, aged forty-two years. Subject to attacks for years. Expectoration greenish and remarkably copious. ℞. Nat. sul. (Tafel), water, three hours. Improvement began after taking a few doses, from which time her expectoration became paler and less abundant; has felt better since than for years and one noteworthy fact is that her expectoration stopped in a few doses, whereas under remedies given in previous attacks it had continued weeks, thus indicating that the Nat. sul. had gotten at the "root of the evil," as Dr. Kent intimated.

